# Effective psychological therapy for PTSD changes the dynamics of specific large‐scale brain networks

**DOI:** 10.1002/hbm.25846

**Published:** 2022-04-08

**Authors:** Marina Charquero‐Ballester, Birgit Kleim, Diego Vidaurre, Christian Ruff, Eloise Stark, Jetro J. Tuulari, Hugh McManners, Yair Bar‐Haim, Linda Bouquillon, Allison Moseley, Steven C. R. Williams, Mark W. Woolrich, Morten L. Kringelbach, Anke Ehlers

**Affiliations:** ^1^ Department of Psychiatry University of Oxford Oxford UK; ^2^ Scars of War Foundation The Queen's College Oxford UK; ^3^ Experimental Psychopathology and Psychotherapy, Department of Psychology University of Zurich Zurich Switzerland; ^4^ Department of Psychiatry, Psychotherapy and Psychosomatics University of Zurich Zurich Switzerland; ^5^ Wellcome Trust Centre for Integrative NeuroImaging, Oxford Centre for Human Brain Activity (OHBA) University of Oxford Oxford UK; ^6^ Zurich Center for Neuroeconomics (ZNE), Department of Economics University of Zurich Zurich Switzerland; ^7^ School of Psychological Sciences Tel Aviv University Tel Aviv Israel; ^8^ Sagol School of Neuroscience Tel Aviv University Tel Aviv Israel; ^9^ Department of Psychology, Institute of Psychiatry, Psychology & Neurosciences King's College London London UK; ^10^ Department of Neuroimaging, Institute of Psychiatry, Psychology & Neurosciences King's College London London UK; ^11^ Centre for Music in the Brain Aarhus University Aarhus Denmark; ^12^ Oxford Centre for Anxiety Disorders and Trauma, Department of Experimental Psychology University of Oxford Oxford UK; ^13^ Oxford Health NHS Foundation Trust Oxford UK

**Keywords:** Cognitive behaviour therapy, Default mode network, fMRI, Hidden Markov Model, PTSD

## Abstract

In posttraumatic stress disorder (PTSD), re‐experiencing of the trauma is a hallmark symptom proposed to emerge from a de‐contextualised trauma memory. Cognitive therapy for PTSD (CT‐PTSD) addresses this de‐contextualisation through different strategies. At the brain level, recent research suggests that the dynamics of specific large‐scale brain networks play an essential role in both the healthy response to a threatening situation and the development of PTSD. However, very little is known about how these dynamics are altered in the disorder and rebalanced after treatment and successful recovery. Using a data‐driven approach and fMRI, we detected recurring large‐scale brain functional states with high temporal precision in a population of healthy trauma‐exposed and PTSD participants before and after successful CT‐PTSD. We estimated the total amount of time that each participant spent on each of the states while being exposed to trauma‐related and neutral pictures. We found that PTSD participants spent less time on two default mode subnetworks involved in different forms of self‐referential processing in contrast to PTSD participants after CT‐PTSD (mtDMN^+^ and dmDMN^+^) and healthy trauma‐exposed controls (only mtDMN^+^). Furthermore, re‐experiencing severity was related to decreased time spent on the default mode subnetwork involved in contextualised retrieval of autobiographical memories, and increased time spent on the salience and visual networks. Overall, our results support the hypothesis that PTSD involves an imbalance in the dynamics of specific large‐scale brain network states involved in self‐referential processes and threat detection, and suggest that successful CT‐PTSD might rebalance this dynamic aspect of brain function.

## 1 INTRODUCTION

Cognitive behaviour therapy (CBT) is one of the first‐line treatments for a very wide range of mental health disorders (e.g. depression, posttraumatic stress disorder (PTSD) and anxiety disorders). Nowadays, such conditions constitute one of the leading causes of disease burden worldwide (Vigo, Thornicroft, & Atun, 2016; Vos et al., 2015). Furthermore, it is estimated that overall treatment response rates averaged across anxiety disorders is approximately 50% at post‐treatment (Loerinc et al., 2015), with about 20–40% of people still meeting criteria for PTSD or depression after CBT (Blanchard et al., 2003; Bradley, Greene, Russ, Dutra, & Westen, 2005; Holtforth et al., 2019). A brain‐level explanation of the effects of CBT in the brain, which is currently missing, is important to understand why this is the case, how to predict who will or will not respond to treatment, and how to help the group of individuals who do not respond.

This study investigated the effects of cognitive therapy for PTSD (CT‐PTSD) (Ehlers, Clark, Hackmann, McManus, & Fennell, 2005), one of the trauma‐focused CBT programmes recommended by international treatment guidelines on the basis of its efficacy (American Psychological Association, 2017; International Society of Traumatic Stress Studies, 2018; National Institute for Health and Care Excellence, 2018). One of the hallmark symptoms of PTSD is the re‐experiencing of the traumatic event (Brewin, Gregory, Lipton, & Burgess, 2010; Keane, Taylor, & Penk, 1997), which can be triggered by a wide range of trauma reminders and, very specifically, carries a feeling of “here and now” (Bar‐Haim, in press; Halligan, Michael, Clark, & Ehlers, 2003; Michael, Ehlers, Halligan, & Clark, 2005). This is thought to indicate decontextualized retrieval of trauma memories (Brewin, 2016; Ehlers & Clark, 2000). The treatment used in this study builds on Ehlers and Clark's (2000) cognitive model of PTSD and includes several specific procedures addressing the disjointed and decontextualised nature of trauma memories as well as problematic appraisals and coping strategies. It includes a specific discrimination training where patients learn to identify triggers of re‐experiencing and to focus on how the present trigger and its current context (“now”) is different from the trauma (“then”). It further addresses excessively negative appraisals of the trauma and/or its sequelae through behavioural experiments, Socratic questioning, providing information and updating the memories for the worst moments of the trauma by linking them with information that makes their meanings less threatening. These strategies rely on the patient's ability to mentalise, that is, to consider the thoughts, beliefs and feelings, both their own and those of other people, that underlie specific behaviours.

At the brain level, recent studies using functional magnetic resonance imaging (fMRI) suggest that three specific large‐scale brain networks and their interactions play an essential role in both the healthy response to acute stress (Hermans et al., 2011; Hermans, Henckens, Joels, & Fernandez, 2014; Van Oort et al., 2017) and the development of a wide range of psychiatric disorders, including PTSD, depression and generalised anxiety (Akiki et al., 2018; Akiki, Averill, & Abdallah, 2017; Menon, 2011). These three large‐scale brain networks are the salience network (essential for cognitive processing of salient stimuli and interoception), the default mode network (DMN, attributed to self‐referential processing) and the central executive network (involved in higher‐order cognitive functions). In particular, acute stress is thought to lead to strategic reallocation of resources towards the salience network, shifting the configuration of these large‐scale brain networks and explaining the behavioural changes observed in stressful situations (Hermans et al., 2011; Van Oort et al., 2017). Further, observed hypoactivation of the DMN in participants who go on to develop PTSD after a traumatic experience has been attributed to the destabilisation caused by a hyperactive or hyperconnected salience network (Akiki et al., 2017; Sripada et al., 2012a).

However, what exactly do we usually mean when we say that a network is hyperactive or hyperconnected? And which tools do we have to quantify changes in reallocation of resources to a particular network? Most PTSD fMRI studies looking at large‐scale networks to date have used “time‐averaged” approaches, in which activation or connectivity (i.e. synchronisation among the regions within a network) is based on fMRI signal averaged over several minutes (Miller et al., 2017; Sripada et al., 2012a). As a result, any derived measures conflate the *amount of time* during which a network is active *(*i.e. *activity time)* or synchronised and the *strength* with which this occurs each time. While this approach is able to access spatial differences between individuals effectively, recent work suggests that it is the variance contained in the activity time of these networks that relates most strongly to affective traits such as fear or anger (Vidaurre, Llera, Smith, & Woolrich, 2019). Furthermore, previous fMRI studies offer support in favour of a more constrained dynamic repertoire in PTSD participants in contrast to healthy controls (Mišić et al., 2016; Ou et al., 2014), which would presumably be reflected in the temporal characteristics of one or more large‐scale brain networks.

Here, we aim to identify the networks showing differences in their activity time(s) in PTSD participants in contrast to trauma‐exposed controls without PTSD, and further hypothesise that symptom reduction through CT‐PTSD will be accompanied by rebalancing of this particular temporal feature. This could potentially shed some light on the processes that lie at the core of PTSD and the specifics of how CBT operates on this disorder at the brain level. To test this hypothesis, we apply a data‐driven approach based on the use of Hidden–Markov models (HMM) (Vidaurre et al., 2016; Vidaurre, Smith, & Woolrich, 2017) on the fMRI data of a treatment‐seeking PTSD population while exposed (1) to pictures of trauma reminders and (2) to neutral pictures. This approach allows us to estimate a (pre‐specified) number of brain networks that recurrently activate across the scanning period—often referred to as brain states in the literature (Stevner et al., 2019; Vidaurre et al., 2017; Vidaurre, Smith, & Woolrich, 2017), and that are shared across our participants. Then, we calculate the overall amount of time during which each participant activates each brain network (i.e. activity time) in two different experimental conditions: trauma‐related or neutral picture presentation. Together with fMRI data, information about the severity of their PTSD symptoms was acquired at each visit. For some participants visits occurred both before and after CT‐PTSD (longitudinal sample) and, for others, only one single visit was completed, either before or after CT‐PTSD. This results in a mixed cross‐sectional and longitudinal design. We refer to participants' data acquired as part of their CT‐PTSD at these two visits as pre‐CT and post‐CT. Some of the treatment‐seeking participants were assigned to a waiting list, in which they were all tested at two points in time—3 months apart on average—without having received any therapy in between (i.e., the pre‐WAIT and post‐WAIT data, serving as control for those collected pre‐CT and post‐CT). Additionally, the same neuroimaging and behavioural data were acquired for a group of trauma‐exposed but healthy participants (referred to as healthy controls) at a single point in time.

Supporting our hypothesis of an imbalance in the dynamics of specific large‐scale brain networks involved in self‐referential processes and threat detection, we found differences between pre‐CT and post‐CT activity times of the two DMN sub‐networks as well as the correlation between PTSD symptomatology and the activity times of the visual ventral stream^+^ and the salience^+^ networks. Interestingly, only the activity time of one of the two DMN sub‐networks, namely the mtDMN^+^, was significantly lower in pre‐CT in contrast to healthy controls, and this also increased up to levels similar to those of healthy controls after successful psychological treatment. Of note, the differences in activity times of the two DMN sub‐networks were context‐specific. The medial temporal DMN, important for contextualised retrieval of autobiographical memories—appeared diminished in PTSD participants in contrast to both healthy controls and post‐CT during the presentation of trauma reminders and in relation to the severity of intrusive memories experienced during the fMRI scan. The activity time of the other DMN—the dorsomedial prefrontal DMN, important for mentalising—appeared diminished in PTSD participants in contrast to post‐CT and showed significant correlations with PTSD symptomatology only during the presentation of neutral pictures. The positive correlations between PTSD symptomatology and the activity times of salience and visual networks appeared specifically during the presentation of trauma reminders.

## 2 MATERIALS AND METHODS

### 2.1 Participants

Participants were treatment‐seeking assault and road traffic accident (RTA) survivors with a primary diagnosis of Posttraumatic Stress Disorder according to DSM‐IV‐TR (American Psychiatric Association, 2000). Inclusion criteria were exposure to an assault or RTA, and a number of standard fMRI‐related safety criteria. They were recruited from an NHS outpatient clinic for anxiety disorders and trauma in London. A trauma‐exposed control group without PTSD who had been exposed to an assault or RTA and fulfilled the fMRI safety requirements was recruited via flyers in the community or via recruitment from ongoing research studies at the Institute of Psychiatry, Psychology and Neuroscience, King's College London, UK. Initial contact was established with 232 individuals, of whom 58 were not interested in taking part in the study, 27 were screened out according to the fMRI safety standards and 58 met other exclusion criteria. Overall, 89 participants were recruited and seven participants dropped out prior to assessment and treatment sessions, could not complete the scan or were not suitable at initial interview. The final sample consisted of 82 participants, of whom 19 were controls, 47 had PTSD at the time of the first scan, and 16 were scanned after recovering from PTSD with a course of CT‐PTSD without having been scanned prior to therapy. Data from eight (four controls and four with PTSD) had to be excluded due to technical problems with their fMRI data. Of the 43 participants with PTSD with valid fMRI data, 31 were assessed before starting CT‐PTSD, and 12 before joining a waitlist; 15 RTA, 28 assault survivors. After a course of CT‐PTSD, 17 were scanned again and data for 14 were analysed as they had recovered from PTSD according to a structured clinical interview PTSD Symptom Scale Interview (PSS‐I) (Foa, Riggs, Dancu, & Rothbaum, 1993) (scored for the PTSD symptom criteria defined in the fourth edition of the Diagnostic and Statistical Manual of Mental Disorders, DSM‐IV, APA 2000). Of the waiting list group, 10/12 returned for the second scan after 3 a month wait, and 8 still met the criteria for PTSD and were analysed.

**FIGURE 1:**
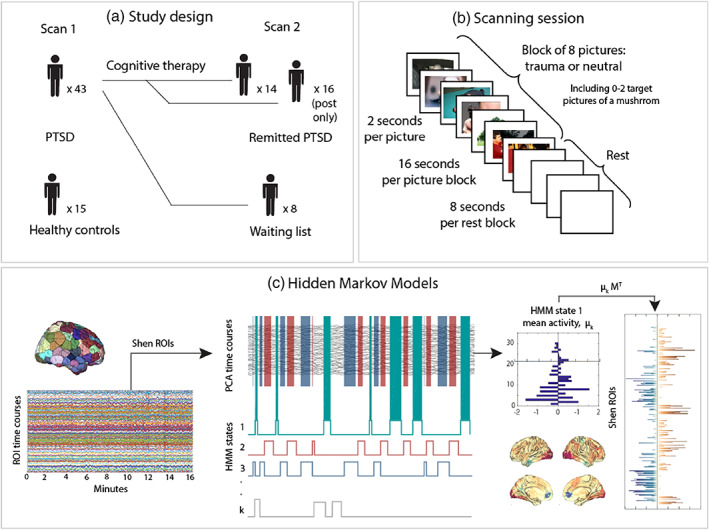
Methods overview. (a) Healthy trauma‐exposed controls (n = 15) and current PTSD participants (*n* = 43; 15 RTA, 28 assault survivors) were scanned at baseline. Participants from the PTSD group were scanned a second time after having received cognitive therapy or being on a waiting list condition (*n* = 8) for approximately 3 months (*n* = 30, *n* = 16 only after therapy). (b) The task used at both scanning timepoints contained blocks of trauma‐related pictures and blocks of neutral pictures. A picture of a mushroom was embedded in each of the blocks 0, 1 or 2 times, to which participants had to react by pressing a button. (c) After fMRI data pre‐processing, a parcellations with 236 regions (Shen, Tokoglu, Papademetris, & Constable, 2013) was applied to extract the timeseries and principal component analysis was performed to reduce dimensionality (75% of variance retained). Subsequently, a Hidden–Markov model was applied, through which we estimated seven different recursive patterns of brain activation and the times at which those patterns dominate brain activity. To visualise each of the patterns, the information was projected back to the Shen space

The total number of scans analysed was 96 (Figure 1a). Participants were assessed and scanned individually either before and after a course of trauma‐focused cognitive therapy for PTSD (Ehlers et al., 2005), before and after waiting to be treated (waiting list), or just at one time point (trauma‐exposed control group without PTSD diagnosis, PTSD patients before or after treatment only). This study design makes possible a mixed longitudinal analysis, including all participants scanned before (*n* = 43) and/or after CT (*n* = 30), as well as a smaller but strictly longitudinal analysis including only participants that attended both visits at the two different time points. The type of therapy provided was always trauma‐focused cognitive therapy for PTSD, irrespectively of whether the participant was scanned only at baseline, only at follow‐up or both at baseline and follow‐up.

Overall, the study included 26 RTA and 48 assault survivors. Average time since the trauma was 4 years and 6.65 months, *SD* = 6.45 years, and the participants mean age was 39.99 years, *SD* = 12.25 years. Some participants were on psychotropic medication, see Supporting Information Table S1.

### 2.2 Procedure

This study was approved by the local ethical review board. Informed consent was obtained from each participant after being given a complete description of the study. Two graduate level psychologists, who were blind to the treatment conditions, conducted structured interviews—the PTSD Symptom Scale Interview (PSS‐I) (Foa et al., 1993) at the first scan and after the treatment or wait list condition. Participants completed the Intrusion Questionnaire for intrusive memories experienced in the scanner (Hackmann, Ehlers, Speckens, & Clark, 2004) Flashback qualities were assessed with the Intrusion Interview (Hackmann et al., 2004) that assesses qualities of intrusive memories that distinguish between intrusive trauma memories in traumatised people with and without PTSD (Michael et al., 2005) (Supporting Information Table S2).

### 2.3 Experimental task

The tasks consisted of 40 blocks of pictures in total, 20 blocks of trauma and 20 blocks of neutral pictures. Order of conditions, that is, trauma versus neutral, was pseudorandomised. Each picture was presented for 2,000 ms followed by an 8 s interval between blocks during which a fixation cross was shown, resulting in a total task duration of 16 min. Each block consisted of 8 pictures, and within each of these series 0–2 mushroom pictures could appear (Figure 1b). They were asked to press a button box every time they detected a target. However, this information was not used for the analysis.

Stimuli were photographs largely derived from the International Affective Picture System (IAPS) (Lang, Bradley, & Cuthbert, 1997), or from public websites, from various categories, that is, trauma survivor, injury, trauma‐related objects, pre‐trauma events (e.g., person sneaking up from behind, a car overtaking in the rain), and pictures depicting actual traumatic events (RTA, assault). Pictures were matched in terms of luminance, number of persons, objects, etc. Parallel categories were introduced for the neutral pictures, that is, person around the house, household‐related objects, two people around the house. Two task versions were constructed, one for assault survivors and one for RTA survivors, with trauma‐specific pictures. Pictures across the task had been rated in pilot studies and matched valence on arousal ratings in order to create two versions of each task for the two assessment times (pre‐ and post‐therapy).

### 2.4 fMRI acquisition and pre‐processing

Brain fMRI scanning was performed on a 1.5 T Siemens Scanner at the Department of Neuroimaging at King's College London. Functional images were acquired using an echoplanar protocol (TR/TE/flip angle: 2400/40/80; FOV: 20× 20 cm; matrix size: 96 × 96) in 36 axial slices (thickness: 3 mm; gap: 0.3 mm). Data pre‐processing was performed using FSL (FMRIB's Software Library, www.fmrib.ox.ac.uk/fsl) and included head motion correction (MCFLIRT), brain extraction, spatial smoothing with a 5 mm FWHM and high‐pass filtering (Gaussian‐weighted least‐squares straight line fitting, with sigma = 50.0 s). Advanced data denoising was carried out using FIX (Griffanti et al., 2014; Salimi‐Khorshidi et al., 2014). Denoised data were first linearly registered to their structural scans and then nonlinearly registered to MNI space. Mean BOLD time‐series were then estimated on 268 brain areas of the Shen functional atlas (Shen et al., 2013) by averaging the BOLD signal over all voxels belonging to each brain area (Figure 1c). From these 268 brain regions, 32 were excluded due to signal drop out. The excluded timeseries belonged to regions of the frontal cortex, cerebellum and temporal cortex. We then ran Principal Components Analysis (PCA) to reduce the data to a number of principal components, explaining 75% of the variance.

### 2.5 Hidden–Markov model analysis

Hidden–Markov modelling is an analysis technique to describe multivariate time series data (in this case extracted through the Shen functional atlas) as a sequence of visits to a finite number of networks, previously validated in both rest and task (Vidaurre, Abeysuriya, et al., 2017). Here, the HMM was estimated on the main principal components of the signal. Each of these networks has a characteristic signature containing both spatial information (i.e., a specific combination of areas showing different degrees of activation relative to the average and forming a spatial map) and temporal information (i.e., a network time‐course showing when these patterns become active). The number of states must be defined beforehand, but the inference of the model can eliminate states if necessary. In this work, we estimated the HMM across a number of states (from *k* = 4 to *k* = 10), computed the free energy for each model in that range of k (Supporting Information Figure S1a), and evaluated their spatial‐maps visually. We chose the model with *k* = 7 on the basis that it was the minimum number of states that provided a separation of the two DMN subnetworks. The run‐to‐run consistency of *k* = 7 was however tested through a comparison of the free energy across 10 initialisations, which showed that the variation of the free energy expressed as a percentage respective to the mean was in the range of −0.0011 and 0.0009 (Supporting Information Figure S2b). Further details regarding the number of states can be found in the Supporting Information. Mathematically, each state is represented by a multivariate Gaussian distribution, which is described by the either the mean or the covariance, or both. In our case, we used only the mean to drive the states, so that each state has a specific average activation, and one single full covariance matrix for the entire sample. Consequently, our model takes into account the intrinsic relationship between brain regions but uses the group‐level covariance (and, therefore, the group‐level functional connectivity). The main differences between our approach and ICA are, then, that the time courses are probabilities instead of signed real numbers, and that the model includes functional connectivity.

The state sequence is modulated by the probability of transition between all pairs of brain states, which is also estimated within the model; that is, the probability of a given state at a specific timepoint *t* depends on the state that was active at the previous timepoint (*t*−1). Here, we applied the HMM on all concatenated scanning sessions, that is including controls at visit 1 and PTSD participants at visit 1 and 2, such that we obtained an estimation of the states at the group level (Figure 1c). This then allowed us to derive subject‐specific information of when a state becomes active, which is referred to as the state time‐course. Based on the state time‐course, we computed the activity time for each state (also referred to as fractional occupancy in related work). These are computed as aggregations of the probabilities of being active at each timepoint for each subject. We derived the activity times independently for each task condition (trauma‐related and neutral blocks of picture presentation). The HMM was inferred using the publicly available HMM‐MAR toolbox, which provides estimates of the parameters of the state distributions, and the probabilities of each state to be active at each time point (Vidaurre, Abeysuriya, et al., 2017; Vidaurre et al., 2016).

### 2.6 Characterisation of states

To make a quantitative assessment of the similarity between our data‐driven networks and the anatomy and function of networks characterised in previous literature, we performed automatic decoding of our spatial maps using the Neurosynth database and decoder (Gorgolewski et al., 2015; Yarkoni, Poldrack, Nichols, Van Essen, & Wager, 2011). Through this approach, we were able to compute the correlations between the spatial maps (above average and below average maps separately), and anatomical and cognitive keywords extracted from over 14.000 fMRI studies (Supporting Information Figure S2). In particular, the 25 terms were retrieved through Neurosynth for each of the above‐ and below‐average activation sub‐components separately. From those, up to 10 cognitive terms showing the strongest correlations were selected to represent each of the networks (Supporting Information S2). The obtained correlation coefficients were moderate in absolute terms, but they were comparable to correlation coefficients obtained in other studies (Gorgolewski et al., 2015; Vidaurre, Smith, & Woolrich, 2017). Results offered support for our initial neuroanatomy‐based labelling of the two DMNs as the mtDMN^+^ and the dmPFC DMN^+^ (for more detail see Supporting Information Figures S2a,b and S3). The remaining five networks were labelled according to the neuroanatomy of their spatial maps as the prefrontal^−^, the salience^+^, the ventral visual stream^+^, the auditory^+^ and the visual/frontal^+^ (Supporting Information Figures S1g and S2c). We then visualised the differences and similarities between the two DMNs, by computing: (1) the area of overlap between the two “above‐average activation” areas, (2) the remaining of mtDMN^+^ minus dmPFC DMN^+^ and (3) the remaining of dmPFC DMN^+^ minus mtDMN^+^.

### 2.7 Statistical analysis

Statistical analyses were run using permutations testing, where we generated surrogate data by permuting the labels of the subjects. First, we looked at differences in the activity times of the main groups (healthy controls, pre‐CT PTSD and post‐CT PTSD) in the entire sample, where the pre‐ and post‐CT PTSD groups contain participants that completed both sessions and participants that were only scanned before or after CT‐PTSD. Note that as a result, some participants were contributing data at a single session and others at both sessions (before and after CT‐PTSD). To assess these differences, we used permutation testing with unpaired t‐tests as the base statistic, that is, ignoring that some of the participants were contributing data at both sessions. For an overview of average symptom severity, see Supporting Information S2.

Then we investigated differences in activity times between PTSD and remitted PTSD only on the subsample of participants who completed both fMRI scans, one before therapy and one after therapy (pre‐CT and post‐CT). As a control for any changes unrelated to therapy, that might occur over time, we also evaluated differences in a different subsample of participants who were scanned at two different points in time but without undergoing any therapy in‐between scans (pre‐WAIT and post‐WAIT). This was done using permutation testing with paired t‐tests as the base statistic. Results are shown both after correcting across multiple comparisons (considering both number of states and of experimental conditions; using False Discovery Rate or FDR (Benjamini & Hochberg, 1995) and before correction for multiple comparisons.

A second statistical analysis was run to test for linear relationships between network activity time and PTSD severity variables. First, we tested for any linear relationships between the activity times of each state and PTSD severity as measured through the three DSM‐IV symptom clusters. Similarly, we tested for any linear relationships between the activity times of each state and (1) number of intrusive memories elicited by the fMRI task, and (2) degree to which these intrusive memories present flashback qualities (i.e. vividness, distress and feeling of “here & now”). Here, in order to integrate different statistical tests into a single *p*‐value, we made use of the non‐parametric combination (NPC) algorithm (Vidaurre, Woolrich, et al., 2018; Winkler et al., 2016); which essentially combines all tests into a single *p*‐value and (since it is family‐wise corrected) avoids the necessity of multiple comparisons within the comprised tests. We integrated the *p*‐values across variables of PTSD symptom severity, an aggregated *p*‐value where the hypothesis is whether at least one of the symptom clusters holds a significant linear relationship with the activity time of a specific state. Similarly, when testing for linear relationships between activity time for each state and the flashback qualities of intrusive memories, we integrated the *p*‐values across number of intrusive memories and the three related variables (i.e., vividness, distress and feeling of “here & now”) using the same procedure. The NPC algorithm to integrate the individual *p*‐values involved a second‐level permutation testing procedure (Winkler et al., 2016). As mentioned, *p*‐values are automatically corrected across multiple comparisons using family‐wise error (FWE) correction (Thomas & Hayasaka, 2003).

## 3 RESULTS

### 3.1 Successful cognitive therapy is associated with longer DMN activity

**FIGURE 2:**
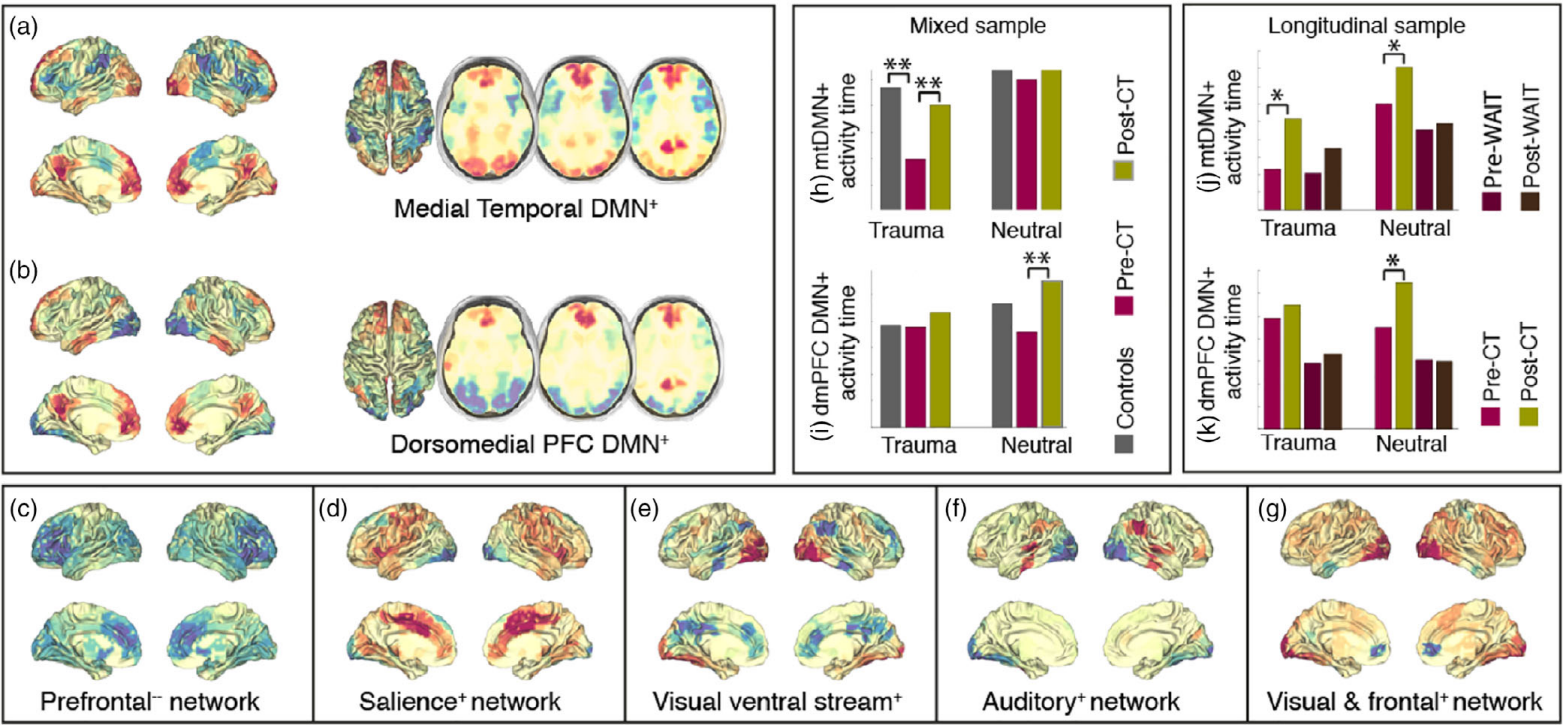
PTSD participants pre‐therapy spend significantly less time activating DMN‐related states in comparison with post‐therapy and healthy controls. (a–g) Spatial maps for our seven networks, labelled according to the areas of above (+) or below (−) average activation. (h) During the presentation of trauma‐related pictures, PTSD participants pre‐CT spend significantly less time activating the medial temporal DMN+ (mtDMN+) than participants post‐CT and healthy controls. (i) During the presentation of neutral pictures, PTSD participants spend significantly less time activating the dorsomedial PFC DMN+ (dmPFC DMN) before than after CT‐PTSD. (j) Increases in the time activity of the mtDMN+ were observed pre‐ and post‐CT for both trauma‐related and neutral pictures (only before correcting across multiple comparisons, *p < .05), **FDR < 0.05 corrected across group comparisons, experimental condition and number of networks

Our analysis of the fMRI data using the HMM resulted in seven differentiated brain states or networks (Figure 2a–g). Each of these networks has a characteristic signature containing both spatial information (i.e., a specific combination of areas showing different degrees of activation relative to the average [+/−]) and temporal information (i.e., a network time‐course showing when these patterns become active). For two of these networks, the spatial map showed areas of above‐average activation (+) consistent with the DMN. More specifically, one of the spatial maps was consistent with the medial temporal DMN (mtDMN^+^) (Figure 2a), and the other one was consistent with the dorsomedial prefrontal DMN (dmPFC DMN^+^) (Figure 2b). The other networks also resemble well‐established functional networks. Details of the assessment of the states' neuroanatomy are described in the methods and in Supporting Information Figures S2 and S3. An overview of the group average activity times for each network, separated for trauma‐related and neutral picture blocks, is presented in Supporting Information Figure S4. Seven was chosen as the minimum number of states that produced a split of the two DMN‐related networks, which we hypothesised to be relevant given previous work (Andrews‐Hanna, 2012; Andrews‐Hanna, Reidler, Sepulcre, Poulin, & Buckner, 2010; Andrews‐Hanna, Smallwood, & Spreng, 2014; Archer, Lee, Qiu, & Annabel Chen, 2015; Northoff & Bermpohl, 2004; Vidaurre, Hunt, et al., 2018).

This analysis included data from the mixed sample: that is, from participants that completed both sessions (before and after CT‐PTSD) as well as participants that completed only one session (before or after CT‐PTSD). The results presented were corrected, using FDR, across group comparisons using, experimental conditions and number of networks. Critically, results show that during the presentation of trauma reminders, pre‐CT participants significantly spent less time activating the mtDMN^+^ than post‐CT participants did (*p* = .0126). We also observed a significant difference in activity time of the mtDMN^+^ between pre‐CT and healthy controls (*p* = .0168) in this condition, but no significant differences were observed between healthy controls and post‐CT (*p* = .7790) (Figure 2h). This pair of significant results suggest that the therapy was successful in bringing activity time of the mtDMN^+^ back to the levels of healthy controls in the post‐CT group when exposed to reminders of the traumatic event. As a sanity check, no significant differences between groups were observed in activity time of the mtDMN^+^ during the presentation of neutral pictures (Figure 2h).

However, during the presentation of neutral pictures, we observed that pre‐CT participants spent significantly less time activating the dmPFC DMN^+^ than post‐CT participants (*p* = .0294) (Figure 2i). No significant differences were observed between healthy controls and any of the PTSD groups, neither pre‐CT nor post‐CT. Also, no significant differences were observed between pre‐ and post‐CT participants in the activity time of the dmPFC DMN^+^ during the presentation of trauma reminders (Figure 2i).

Hence, activity times of both the mtDMN^+^ and the dmPFC DMN^+^ differ between groups, but these differences depend on the experimental condition. Specifically, differences in activity time of the mtDMN^+^ are specific to the blocks during which trauma reminders are presented, while differences in activity time of the dmPFC DMN^+^ appeared only during the presentation of neutral pictures.

### 3.2 Changes observed in activity time of the DMN after CT were not present in the waiting list group

We then ran a second analysis to investigate whether the increased activity time of the DMN networks observed in participants after CT was present in the group of participants that were assigned to a waiting list condition (Figure 2j,k). This was a strictly longitudinal analysis, including only participants that had completed both scans: a first scan at baseline, followed by a second scan either after CT‐PTSD (*n* = 14) or after an average of 3 months on the waiting list (*n* = 8). Results presented here are corrected across group comparisons, experimental conditions and number of networks; in cases in which significance was borderline, we also show uncorrected results. Findings of increased activity time of the mtDMN^+^ in the post‐CT during the presentation of trauma‐related pictures were corroborated, although they were only borderline significant after correction for multiple comparisons (pre‐CT vs. post‐CT, *p* = .0525 after correction, *p* = .008 uncorrected). In addition to our cross‐sectional results, in this longitudinal analysis, we also observed an increase in the activity time of the mtDMN^+^ in the post‐CT group during the presentation of neutral pictures before correction for multiple comparisons (pre‐CT vs. post‐CT, *p* = .242 after correction, *p* = .0350 uncorrected) (Figure 2j). However, no significant changes in activity time of the mtDMN^+^ were observed when comparing the scan obtained at baseline to the scan obtained after 3 months of waiting (pre‐WAIT vs. post‐WAIT), neither during the presentation of trauma‐related (*p* = .3629) nor during the presentation of neutral pictures (*p* = .40).

In relation to activity time of the dmPFC DMN^+^ during the presentation of neutral pictures, we observed that the waiting list group did not show any differences across sessions (*p* = .48), and the CT group showed only a non‐significant increase in the time visiting the dmPFC DMN^+^ after CT once we had corrected for multiple comparisons (*p* = .0952 after correction, *p* = .0136 uncorrected) (Figure 2k).

No significant changes between the first and the second visit were found in the activity time of any of the other networks, neither after CT nor after waiting list.

To test for a possible interaction between treatment condition and time, we calculated the “amount of change” in activity time for the mtDMN^+^ and the dmPFC DMN^+^ between the two visits, both for the pre‐CT versus post‐CT and pre‐WAIT versus post‐WAIT conditions. We then compared these values to assess whether there were any significant differences in the amount of change in the activity time of the two DMN networks linked to the treatment condition (CT‐PTSD vs. Waiting list). This analysis yielded no significant results.

### 3.3 Activity time of DMN and salience networks correlate with PTSD severity

To further investigate the association between network activity times and PTSD symptomatology, we computed correlations between the activity times of each of the networks and the severity of each of the three PTSD symptom clusters (i.e. Re‐experiencing, Avoidance and Hyperarousal) as described in the DSM‐IV (American Psychiatric Association, 2000). This was done in order to investigate whether time spent on any of the networks was related to any specific symptom clusters. We integrated different statistical tests into a single *p*‐value using the non‐parametric combination (NPC) algorithm (Vidaurre, Woolrich, et al., 2018; Winkler et al., 2016), which integrates all tests into a single *p*‐value. In particular, we aggregated *p*‐values across symptom clusters (instead of across networks) in order to assess whether at least one of these symptom clusters bears a relationship to the activity time of any of the networks. Additionally, we also considered individual correlations corrected across multiple comparisons. As with previous analyses, we did this separately for the neutral and the trauma‐related conditions. The analysis included all participants from the therapy (pre‐ and post‐CT) and the waiting list groups, excluding only healthy controls (*n* = 81).

**FIGURE 3:**
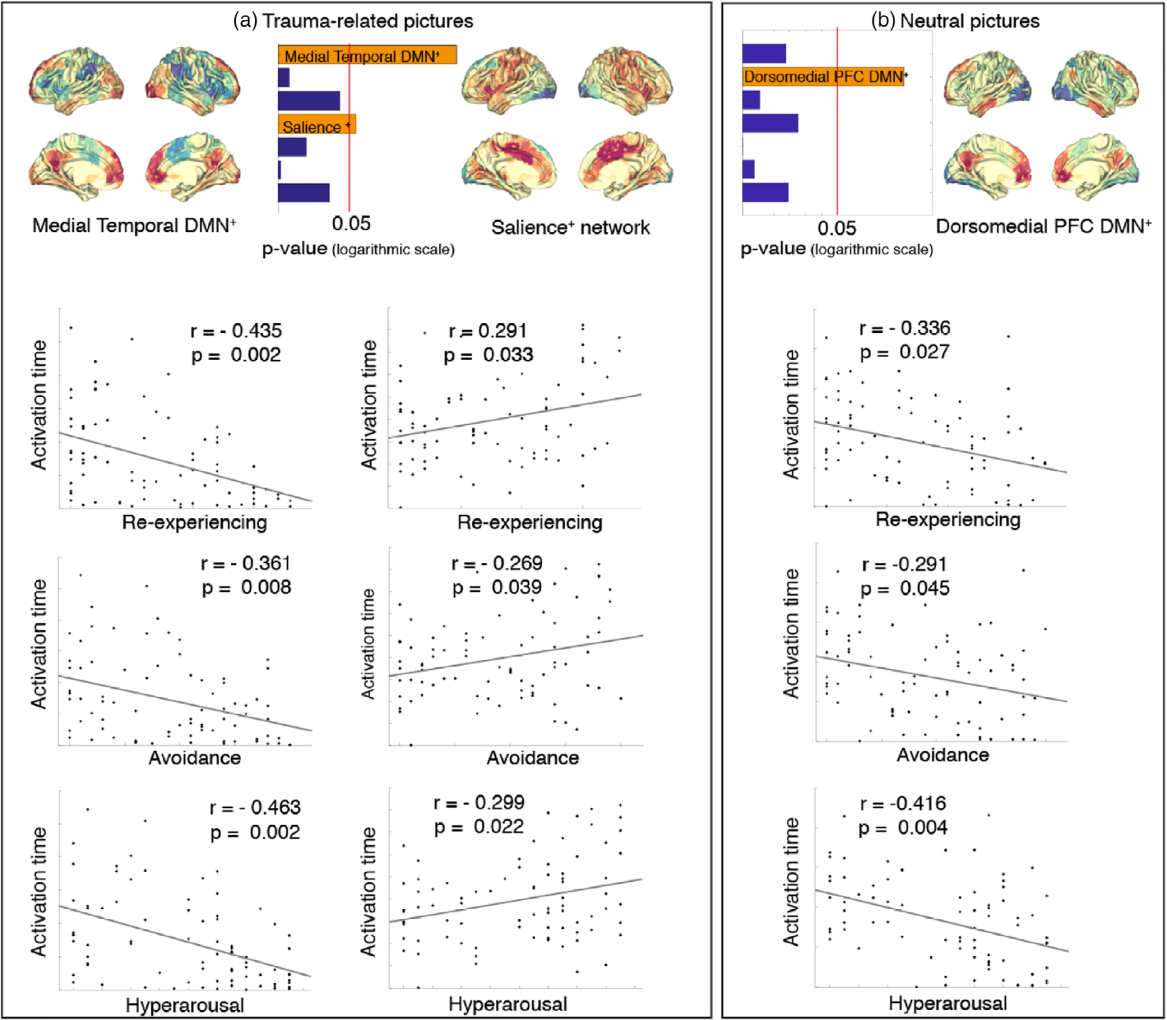
Symptom severity shows significant correlations with activity time of the sub‐networks of the DMN, the salience network and each of the three symptom clusters (re‐experiencing, avoidance and hyperarousal). (a) Integration of different statistical tests into a single *p*‐value through non‐parametric combination algorithm reveals a significant relationship between PTSD symptoms and the activity times of the medial temporal DMN^+^ and the salience^+^ networks. More in detail, the first column shows the significant negative correlation between the activity time of the medial temporal DMN (mtDMN^+^) and re‐experiencing (*p* < .002), avoidance (*p* < .008) and hyperarousal (p < .002), while the second column shows the positive correlation with the activity time of the salience+ state and each of the symptom clusters: re‐experiencing (*p* < .033), avoidance (*p* < .039) and hyperarousal (*p* < .002). (b) Statistical integration into a single *p*‐value shows a significant relationship between PTSD symptoms and the activity times of the dorsomedial PFC DMN^+^ (dmPFC DMN+) . The third column shows the significant negative correlation between the activity time of the dmPFC DMN^+^ and re‐experiencing (*p* < .027), avoidance (*p* < .045) and hyperarousal (*p* < .004)

We found that the activity times of the mtDMN^+^ and the salience^+^ networks were related to at least one of the symptoms clusters (*p* = .006 for mtDMN^+^ and *p* = .0399 for the salience^+^ network, respectively) during the presentation of trauma‐related pictures (Figure 3a). Also, the activity time of the dmPFC DMN^+^ was associated with at least one of the symptoms clusters (*p* = .0046) (Figure 3b) during the presentation of neutral pictures. Closer examination revealed that each of the symptom clusters significantly correlated with the activity times of the mtDMN^+^ (*re‐experiencing (p < .002)*, *avoidance (p < .008) and hyperarousal (p < .002))* (Figure 3, column 1), dmPFC DMN^+^ (*re‐experiencing (p < 0.027)*, *avoidance (p < .045) and hyperarousal (p < .004))* (Figure 3, column 3), and salience^+^ networks (*re‐experiencing (p < .033)*, *avoidance (p < .039) and hyperarousal (p < .022))* (Figure 3, column 2), after correcting across number of networks and number of symptom clusters.

Furthermore, cluster‐specific positive correlations during the presentation of trauma‐related pictures were found between the activity time of the prefrontal^−^ network and re‐experiencing symptoms (*p* = .024) as well as between the visual/frontal^+^ and hyperarousal (*p* = .03). Activity time from the other brain networks did not show any significant relationship with PTSD symptomatology after correcting across number of networks and number of symptom clusters.

### 3.4 The flashback‐like qualities of intrusive memories in the scanner correlate with activity time of several brain networks

We explored the relationship between activity times and the flashback qualities (i.e. vividness, distress and feeling of “here & now”) associated with intrusive memories characteristic of PTSD. When intrusive memories arise, the re‐experiencing of the traumatic event occurs in a very vivid way, mostly consisting of visual impressions, and carries a feeling of the event occurring in the present moment with the consequent distress (Ehlers & Clark, 2000; Michael et al., 2005).

**FIGURE 4:**
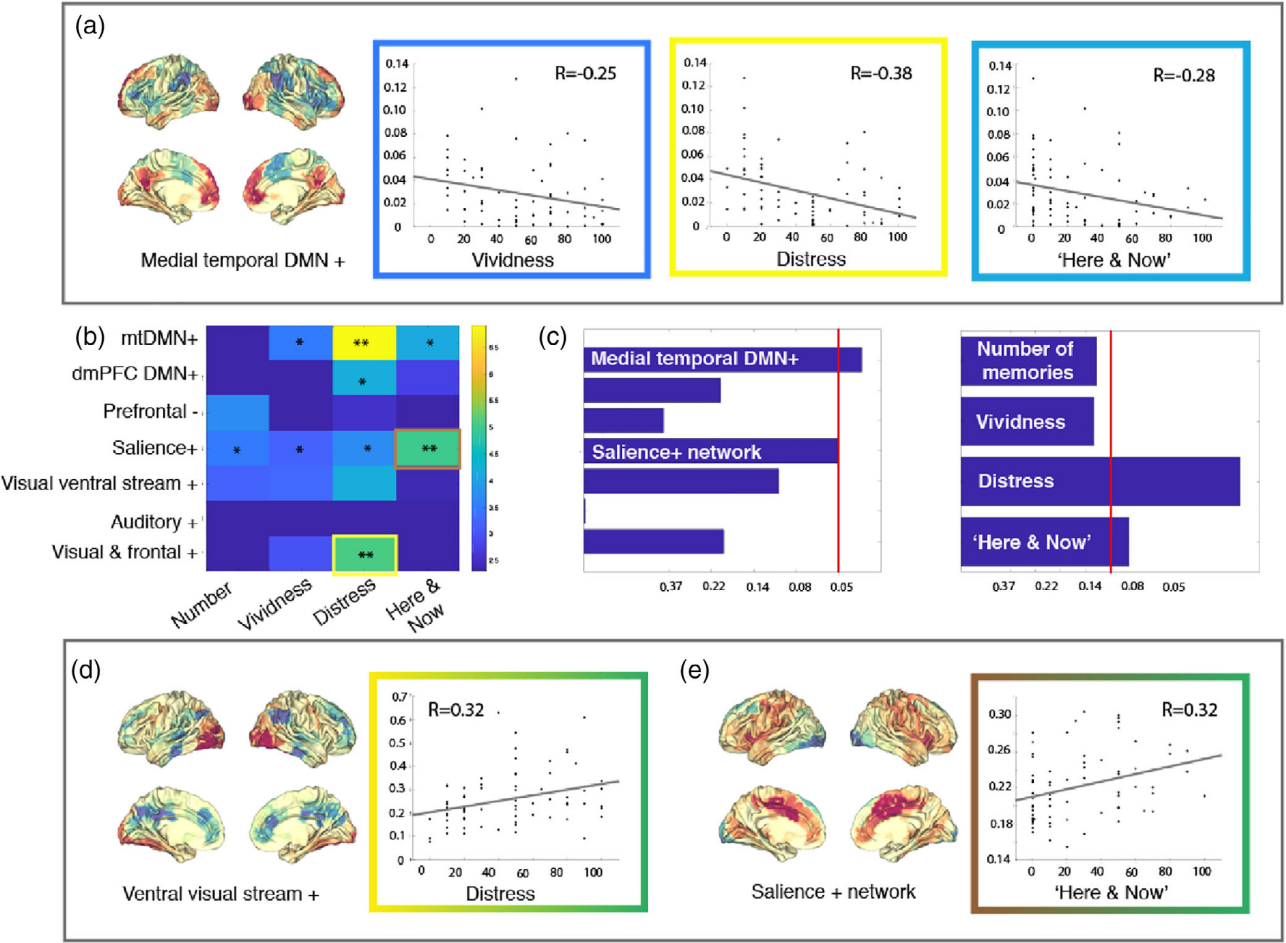
The activity time of different states is significantly correlated with the flashback‐like qualities of intrusive memories in the scanner. (a) Correlation between time spent in the medial temporal DMN^+^, and quality of the intrusive memories. All variables related to the quality of the memory are negatively correlated to time spent on this network, although only the relationship with how distressing the intrusive memories were remains significant after FWER correction. (b) Matrix of correlation *p*‐values for each of the network activity times with each of the memory variables (*p < .05, **FWER < 0.05). (c) Aggregated *p*‐values for the correlations of networks' activity times across memory variables (left) and memory variables across networks' activity times (right), FWER‐corrected. Time spent in the Visual ventral stream^+^ (d) and Salience^+^ networks (e) correlates with how distressing and how much the memory felt as if it was currently happening, respectively

Analyses were carried out on the subsample of participants who experienced at least one intrusive memory during the fMRI task, which included not only PTSD participants before and after CT but also six healthy controls that had a flashback while in the scanner (*n* = 72). Again we considered aggregated statistical tests into a single *p*‐value through the NPC algorithm (Vidaurre, Woolrich, et al., 2018; Winkler et al., 2016), as well as individual correlations corrected across multiple comparisons. Results from correlations aggregated across flashback‐like qualities revealed that the activity times of mtDMN^+^ and the salience network hold linear relationships with at least one of the flashback‐like qualities (Figure 4c, left). Individual correlations between the activity time of each of the networks and each of the flashback‐like qualities revealed a negative correlation between mtDMN^+^ and memory‐related distress (*p* = .036 corrected) and a positive correlation between the salience^+^ and the feeling of “here & now” (*p* = .0607 after correction, *p* = .0061 uncorrected). These were the only significant correlations after correction for multiple comparisons between the mtDMN^+^ and the salience networks, and specific flashback‐like qualities. However, other qualities were significant before correction for multiple comparisons (i.e., vividness and the feeling of “here & now” for the mtDMN^+^ and all other three variables for the salience network). Furthermore, we observed a positive correlation between the activity time of the ventral visual stream^+^ and distress, which was significant after correction for multiple comparisons (Figure 4d).

Further, we computed aggregated *p*‐values across the seven networks for each of the flashback‐like memory variables. Here, we observed that the degree of distress caused by the memory, and how much it made the participant feel as if it was happening again (“here & now”), could be predicted by a combination of the activity times of the estimated networks (Figure 4c, right).

### 3.5 There are significant differences in the amount of transitions between the mtDMN^+^ and the dmPFC DMN^+^ before and after cognitive therapy, but not before and after being on a waiting list

Analyses investigating differences in the transitions between states for the mixed sample show that there were no significant differences when comparing controls versus PTSD, PTSD versus Remitted and controls versus remitted (Supporting Information Figure S5). However, the same analysis on the longitudinal sample revealed that there is a significant difference in the number of transitions between the mtDMN^+^ and the dmPFC DMN^+^ before and after cognitive therapy, but not before and after being on a waiting list (Supporting Information S6). However, the small longitudinal sample size calls for caution when interpreting these results.

## 4 DISCUSSION

This study suggests that successful CT‐PTSD is associated with changes in the time spent in specific large‐scale brain networks. We found that participants with PTSD spent significantly less time engaging two different DMN‐related networks than did healthy participants: one network with a stronger temporal component (mtDMN^+^) and one with a stronger dorsomedial prefrontal component (dmPFC DMN^+^). Furthermore, the time these two networks were active in PTSD increased after successful CT to levels similar to those of healthy participants. In line with these results, we found a significant negative correlation between severity of PTSD symptoms and time activating these DMN‐related networks. This resonates with previous studies of DMN hypoactivity and hypoconnectivity in PTSD(Bluhm et al., 2009; King et al., 2016; Lanius et al., 2010; Sripada et al., 2012b). Our results further add to this literature by suggesting that activity time may be one of the network behaviours playing a role in the observed dysfunction in the DMN, and that this aspect was modified after successful CT‐PTSD.

The data‐driven estimation of two DMN networks is consistent with empirical work and related theories that consider the DMN a constellation of several subsystems associated with different forms of self‐referential processing (Andrews‐Hanna, 2012; Andrews‐Hanna et al., 2010; Andrews‐Hanna et al., 2014; Archer et al., 2015; Northoff & Bermpohl, 2004; Vidaurre, Hunt, et al., 2018). Beyond normalisation of the two DMNs activity time after CT‐PTSD, our study shows spatial specificity within the DMN in relation to the experimental condition (trauma vs. neutral picture presentation) and the severity of intrusive memories in the scanner. In particular, we found that the strength of intrusive memories with flashback‐like qualities, the hallmark symptom of PTSD, was only related to the activity time of the mtDMN^+^ during the presentation of trauma‐related pictures, but not of the dmPFC DMN^+^ (Figure 4a). From the two networks, only the spatial map of the mtDMN^+^ showed above‐average activation in parahippocampal cortex, hippocampal formation and visual cortex. Together with the midline structures (i.e., medial prefrontal cortex and precuneus), whose above‐average activation was part of both DMN networks, the hippocampal and parahippocampal formations form a network that has been related to the contextualised retrieval of episodic memories (Andrews‐Hanna et al., 2010; Andrews‐Hanna, Smallwood, & Spreng, 2014; Cabeza & St Jacques, 2007; Ranganath & Ritchey, 2012; Rugg & Vilberg, 2013). Furthermore, changes in time‐averaged functional connectivity have been previously observed among some of these regions following treatment for PTSD for example, (Zhu et al., 2018).

In the context of the cognitive theory of PTSD (Ehlers & Clark, 2000), one of two factors resulting in the sense of current threat that perpetuates PTSD symptomatology is the spatially and temporally decontextualised memory of the traumatic event. While more research is needed in order to establish a direct link between the decontextualized memory and changes to mtDMN^+^ dynamics, we take these as initial findings suggesting that difficulties in activating this network during the presentation of trauma reminders in PTSD might be related to decontextualized retrieval of traumatic memories. In line with this hypothesis, a previous study by St Jacques and colleagues (St. Jacques, Kragel, & Rubin, 2013) on trauma‐naïve and PTSD participants showed that participants who experienced memory retrieval of the traumatic event from the perspective of their own eyes (as opposed to experiencing it in a detached way from oneself) recruited the medial temporal lobe network more.

From a system neuroscience point of view, it has been argued that deficient encoding of the traumatic event prevents migration of the memory from the hippocampal formation to a distributed network of brain regions during the consolidation process (Mcclelland, Mcnaughton, & Reilly, 1995; Squire & Alvarez, 1995). This “earlier” form of the memory remains under‐contextualised and, therefore, indiscriminately accessible; that is, an intrusive memory with flashback‐like qualities. One of the goals of CT‐PTSD is the development of a coherent narrative that places the series of events in chronological context and includes the more up‐to‐date information that the patient has now (e.g. “I did not die” or “It was not my fault”). This is thought to reduce re‐experiencing through the creation of an updated, contextualised memory that inhibits the cue‐driven retrieval of intrusive memoires and is experienced as an event in the past rather than the present (Conway, 1997; Ehlers & Clark, 2000). CT‐PTSD focuses specifically on the worst moments of the trauma, which are represented in re‐experiencing symptoms, and targets those parts of the trauma memory through a process of contextualising and updating. Our findings increased activity time of the mtDMN^+^ during the presentation of trauma reminders after CT‐PTSD adds a new piece to the puzzle of the cognitive and neural processes underlying PTSD recovery.

Continuing with the cognitive theory of PTSD, the other main factor contributing to the sense of current threat is the presence of negative appraisals of the traumatic event (e.g., “What happened was my fault” or “My family despises me because of what I did/didn't do”) (Ehlers & Clark, 2000). CT‐PTSD identifies those appraisals and changes them through a variety of techniques that use our mentalisation processes (Björgvinsson & Hart, 2006); that is, our ability to understand the mental states that underlie our own and others' overt behaviour. Specifically, the dmPFC DMN^+^ is thought to support mentalisation (Andrews‐Hanna et al., 2010, 2014). While our findings are still preliminary, one possibility is that the observed increase in activity time of this network after CT‐PTSD might be related to the work done to change negative appraisals during the psychological therapy.

Beyond the disruption of the DMN, previous neuroimaging literature links hyperactivity and hyperconnectivity of the salience network to PTSD (Abdallah et al., 2019; Sripada et al., 2012b). Our results are in line with these findings, as we show positive correlations between activity time of a salience network and severity of PTSD symptomatology. We show this in terms of the DSM‐IV symptom clusters (Figure 3), and also flashback qualities of intrusive memories (Figure 4) during the presentation of trauma‐related pictures. Other networks with a strong visual component also show positive relationships between their activity time during the presentation of trauma‐related pictures and symptom severity. Overall, we could interpret this as a result of increased saliency of trauma‐related images for people with PTSD, accompanied by an increase in alertness that might lead to more frequent activity of perception and salience networks.

By contrast to studies that average the brain signal across the temporal dimension, the higher temporal afforded by the HMM enables exploration of new questions regarding the stress response and psychiatric disorders. Recent theories based on animal research propose that the stress response entails dynamic shifts in network balance, with the DMN, the salience and the central executive networks playing the key roles (Hermans et al., 2011; Hermans et al., 2014; Van Oort et al., 2017). It has been further argued that some affective disorders such as PTSD are likely linked to disruptions in the shifts of these large‐scale networks (Akiki et al., 2017; Menon, 2011), which could perhaps appear as an inability to disengage a specific pattern of brain activity and transition into another one. More recent work has shown that the variance contained in the fluctuations of these networks is more strongly related to affective traits such as fear or anger than the individual differences found in connectivity measures derived from the totality of the fMRI signal averaged over several minutes (Vidaurre et al., 2019). While much has been hypothesised about the dynamic behaviour of large‐scale brain networks, appropriate tools to address these hypotheses directly in human brain data were underdeveloped. The data‐driven approach used here, by allowing for concurrent estimation of both large‐scale brain networks and the specific time periods during which these dominate brain activity, was able to suggest specific time difficulties in engaging DMN components in participants with PTSD.

PTSD is a useful model for studying the neural changes that occur in the brain following CBT. While the focus of this work is on PTSD, it is important to note that PTSD shares many commonalities with other psychiatric disorders, such as depression or anxiety disorders (Brady, Killeen, Brewerton, & Lucerini, 2000; Gros, Price, Magruder, & Frueh, 2012). These disorders can also develop after a traumatic experience, are highly comorbid with PTSD, and are often treated through a range of CBT programmes (National Institute for Health and Care Excellence, 2009, 2011). At the brain level, these disorders appear to have some common neurobiological signatures (Etkin & Wager, 2007) and share some aspects of the alterations that occur in the dynamic shift of large‐scale brain networks (Menon, 2011). Here, we explored the brain changes pre‐ and post‐CT in a population of participants with PTSD, some of whom have comorbid depression and anxiety disorders. Our results, therefore, might be relevant to overarching processes important for regaining mental health through CT, going beyond the specifics of PTSD psychopathology.

There are some limitations to the present work. First, the sample sizes are not equal; even though our statistical tests are not biassed by this fact and the HMM was run without any information of the groups, the HMM might describe the largest group (participants with PTSD) more precisely than the others. Furthermore, a larger longitudinal sample would be desirable in order to reach more robust conclusions related to the changes in activity time of the networks in the pre‐ and post‐CT and pre‐ and post‐waiting list conditions. Second, the time spent engaging the mtDMN^+^ during the presentation of trauma‐related pictures in the mixed analysis (Figure 1h) was partly explained by a combination of differences in gender and medication when comparing healthy controls to pre‐CT participants. However, this was not the case for pre‐CT versus post‐CT, where the differences persisted even after controlling for these two variables. While in an ideal scenario all groups should be matched for all potentially confounding variables, this is not always possible—future studies should still aim to reduce these differences. Third, while we decided to focus on the trauma‐related and neutral blocks of pictures presented, this is a simplification of our experimental task, which further included the presentation of 0–2 mushroom pictures per block. The present work does not consider the potential influence of the mushroom picture presentation in the activity times of the networks; this remains to be explored in future work. Fourth, our interpretation of the data‐driven large‐scale brain networks was supported by an automatic meta‐analysis carried out through Neurosynth (Yarkoni et al., 2011) (Supporting Information Figures S2 and S3). While we think that this has the potential to provide useful insights and to foster interesting questions for further research, we are also aware of the limitations of reverse inference based on literature mining. Fifth, from a methodological point of view, the use of PCA can degrade the quality of an HMM estimation (Vidaurre, 2021), but this does not affect the statistical validity of our results.

Overall this study shows that time‐varying approaches may allow us to test some core aspects of cognitive and biological models of psychiatric disorders. Future studies could address the specifics of direct interactions between large‐scale brain networks, in particular the salience and the DMN, to characterise further the dynamics related to PTSD symptomatology. Studies including treatment‐resistant cases in their sample might be able to clarify how their dynamics differ from those who respond to treatment, and whether this is most strongly related to the activity time or the activation strength of specific large‐scale brain networks. In due course, this could lead to a more precise understanding of suboptimal mechanisms in treatment‐resistant cases, and the subsequent optimisation of interventions.

## Supporting information


**Supplementary Table 1.** Demographic information for all groups
**Supplementary table 2.** PTSD symptomatology
**Supplementary table 3.** P‐values obtained through permutation testing for differences in demographic variables and symptom severity
**Supplementary figure 1.** Assessment of k through measures of free energy
**Supplementary figure 2.** Correspondence between cognitive terms found in the literature and the areas of above (+/red) and below‐average activation (−/blue) for each of the seven networks
**Supplementary figure 3.** Isolation of non‐overlapping areas between the two DMNs suggests functional differentiation of their predominant cognitive processes
**Supplementary figure 4.** Healthy controls and patients with PTSD before and after CT spend different amounts of time on each network in the trauma‐related and neutral conditions
**Supplementary figure 5.** No significant differences were observed between controls, PTSD and remitted participants in states transitions
**Supplementary figure 6.** Differences in the number of transitions between DMN subnetworks are observable before and after CT, but not before and after being on a waiting list

## Data Availability

The analysis leveraged the HMM‐MAR toolbox, publicly available (https://github.com/OHBA-analysis/HMM-MAR). The imaging and clinical data that support the findings of this study are not publicly available to protect the privacy of research participants in accordance with good clinical practice.
