# Supplementary material for: Effective psychological therapy for PTSD changes the dynamics of specific large‐scale brain networks
Source: Hum Brain Mapp. 2022 Apr 8;43(10):3207–20. doi: 10.1002/hbm.25846 (PMC9188968; doi:10.1002/hbm.25846)
Supplement: Supplementary file 1 — Supplementary Table 1. Demographic information for all groups Supplementary table 2. PTSD symptomatology Supplementary table 3. P‐values obtained through permutation testing for differences in demographic variables and symptom severity Supplementary figure 1. Assessment of k through measures of free energy Supplementary figure 2. Correspondence between cognitive terms found in the literature and the areas of above (+/red) and below‐average activation (−/blue) for each of the seven networks Supplementary figure 3. Isolation of non‐overlapping areas between the two DMNs suggests functional differentiation of their predominant cognitive processes Supplementary figure 4. Healthy controls and patients with PTSD before and after CT spend different amounts of time on each network in the trauma‐related and neutral conditions Supplementary figure 5. No significant differences were observed between controls, PTSD and remitted participants in states transitions Supplementary figure 6. Differences in the number of transitions between DMN subnetworks are observable before and after CT, but not before and after being on a waiting list [file HBM-43-3207-s001.docx]

**Supplementary information**

|  | **Mixed sample for group comparison** | | | **Longitudinal sample** | | | |
| --- | --- | --- | --- | --- | --- | --- | --- |
|  | Controls (n=15) | PTSD  (n=43) | Remitted from PTSD  (n=30) | preCT  (n=14) | postCT  (n=14) | preWAIT  (n=8) | postWAIT  (n=8) |
| Age  (mean and SD) | 38.11/12.12 | 37.32/12.48 | 38.23/12.55 | 37.21/14.06 | 37.77/13.98 | 42.26/13.26 | 43.43/13.67 |
| Gender (M/F) | 11/4 | 17/26 | 16/14 | 8/6 |  | 3/5 |  |
| Years since trauma  (mean and SD) | 4.48/6.18 | 4.21/6.87 | 3.35/4.12 | 4.33/4.85 |  | 6.59/9.42 |  |
| Trauma type (RTA/Assault) | 4/11 | 15/28 | 11/19 | 4/10 |  | 3/5 |  |
| Medication (SNRI, SSRI, beta blockers, tricyclic antidepressants) | 1 (6.7%) | 12 (27.9%) | 8 (26.7%) | 6 (42.9%) | 6 (42.9%) | 1 (12.5%) | 1 (12.5%) |

***Supplementary Table 1. Demographic information for all groups.***

|  | **Mixed sample for group comparison** | | | **Longitudinal sample** | | | |
| --- | --- | --- | --- | --- | --- | --- | --- |
|  | Controls (n=15) | PTSD  (n=43) | Remitted from PTSD  (n=30) | preCT  (n=14) | postCT  (n=14) | preWAIT  (n=8) | postWAIT  (n=8) |
| PSSI re-experiencing  (mean and SD) | 0/0 | 11.10/4.02 | 1.23/2.37 | 10.29/3.77 | 1/1.04 | 11.63/4.64 | 8.88/4.91 |
| PSSI avoidance | 0.53/1.19 | 13.01/4.02 | 2.2/2.37 | 11.64/3.30 | 2/2.72 | 13.44/4.17 | 11.63/4.41 |
| PSSI arousal | 0.53/1.41 | 11.16/2.57 | 2.21/2.41 | 11.07/1.44 | 2.29/2.49 | 9.75/4.17 | 11.38/3.58 |
| PSSI total | 1.07/2.49 | 35.33/8.43 | 5.59/11.14 | 33/6.36 | 5.29/5.36 | 34.81/12.01 | 31.88/11.14 |
| Percentage of memories in scanner | 5.67/3.61 | 37.87/28.30 | 25.22/26.34 | 32/27.0 | 7.31/7.11 | 28.75/12.46 | 22.86/13.80 |
| Vividness | 35/35.64 | 58.55/25.25 | 37.83/29.38 | 59.58/28.64 | 26.25/31.59 | 58.13/23.29 | 68.57/23.40 |
| Distress | 10/6.32 | 56.84/25.35 | 27.82/28.75 | 57.5/29.58 | 11.25/11.26 | 53.75/25.04 | 62.86/29.84 |
| ‘Here /Now’ | 1.67/4.08 | 34.76/25.90 | 12.61/27.17 | 27.92/31.58 | 2.5/4.63 | 35/23.90 | 45.71/22.99 |

***Supplementary table 2. PTSD symptomatology***

|  | **Controls vs. PTSD** | **PTSD vs. Remitted from PTSD** | **Controls vs. Remitted** | **Pre-CT vs Post-CT** | **Pre-WAIT vs. Post-WAIT** |
| --- | --- | --- | --- | --- | --- |
| Age | p=0.50 | p=0.49 | p=0.50 | - | - |
| Years since trauma | p=0.45 | p=0.34 | P=0.41 | - | - |
| PSSI re-experiencing | p=0.0006 | p=0.10 | p=0.0004 | p=0 | p=0.0340 |
| PSSI avoidance | p=0.0001 | p=0.1646 | p=0.0014 | p=0.0001 | p=0.0720 |
| PSSI arousal | p=0.0001 | p=0.0006 | p=0.0253 | p=0.0002 | p=0.8820 |
| PSSI total | p=0.0001 | p=0.0783 | p=0.0001 | p=0.0002 | p=0.1462 |
| Percentage of memories in scanner | p=0.0270 | p=0.0824 | p=0.1707 | p=0.014 | p=0.0559 |
| Vividness | p=0.1403 | p=0.0030 | p=0.3349 | p=0.0298 | p=0.7724 |
| Distress | p=0.0004 | p=0.0004 | p=0.2180 | p=0.0163 | p=0.7395 |
| ‘Here /Now’ | p=0.0003 | p=0.2459 | p=0.0008 | p=0.1886 | p=0.8139 |

***Supplementary table 3. P-values obtained through permutation testing for differences in demographic variables and symptom severity.***

**
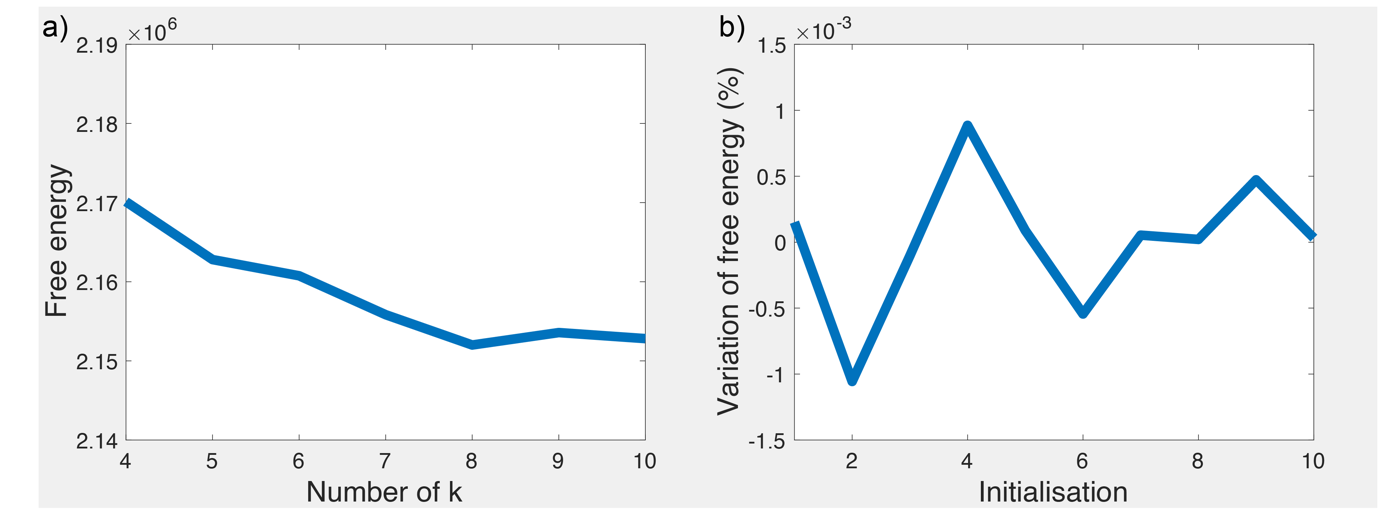
**

***Supplementary figure 1. Assessment of k through measures of free energy*** ***a)*** *The computation of free energy for k=4 to k=10 shows that the free energy is minimal with fe_k8_= 2152 x10^6^, but the difference with models that have 7 or more states can be considered small (fe_k7_ = 2.156 x10^6^, fe_k9_=2.154 x10^6^, fe_k10_ = 2.153 x10^6^).* ***b)*** *Comparison of the free energy across 10 initialisations for k=7 to assess the run-to-run consistency showed that its variation expressed as a percentage respective to the mean was in the range of -0.0011 and 0.0009.*

**
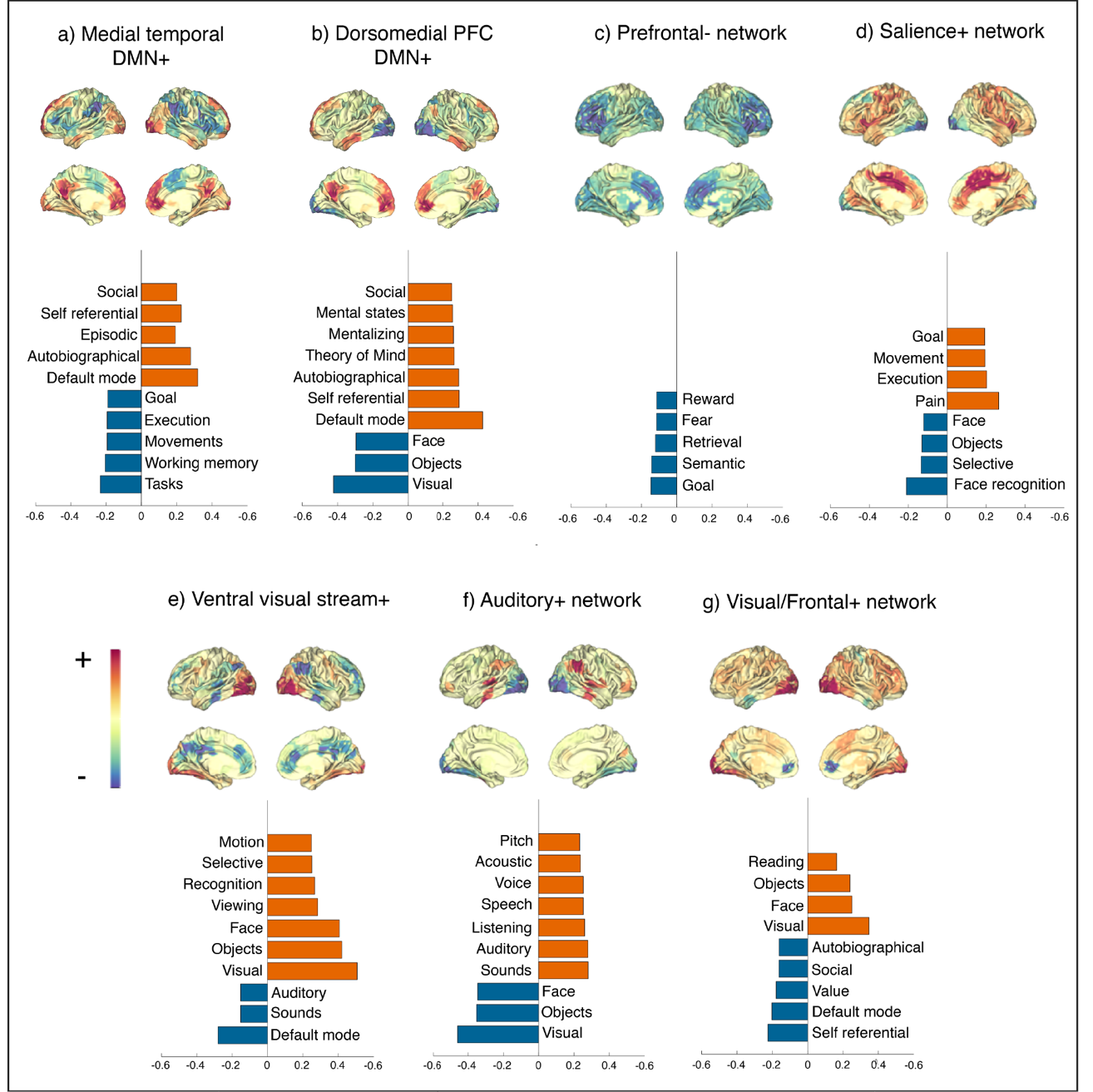
**

##### **Supplementary figure 2. Correspondence between cognitive terms found in the literature and the areas of above (+/red) and below-average activation (-/blue) for each of the seven networks.** Automatic decoding was performed through Neurosynth (Yarkoni et al., 2011). Up to 10 cognitive terms most strongly correlating with the spatial maps (a-g) are shown below each of the networks.

***
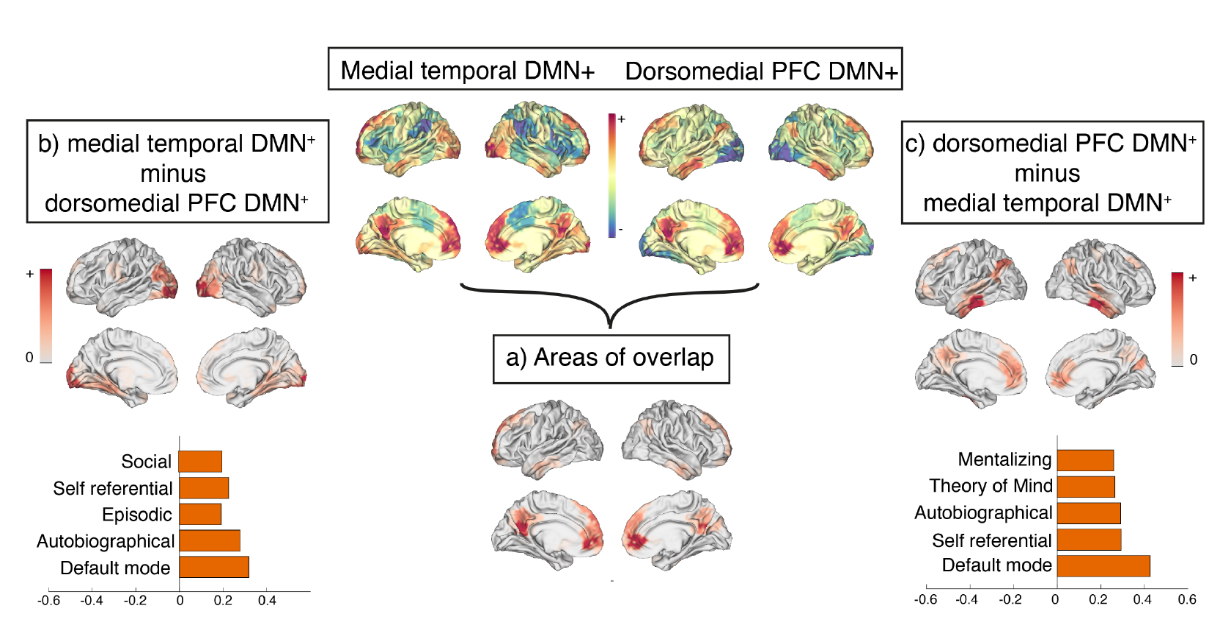
Supplementary figure 3. Isolation of non-overlapping areas between the two DMNs suggests functional differentiation of their predominant cognitive processes.*** *a)* *Areas of overlap involve mainly the anterior medial prefrontal cortex and the posterior cingulate cortex. b) Subtraction of the dmPFC DMN^+^ from the mtDMN^+^ reveals remaining above-average activation in the visual, retrosplenial and parahippocampal cortices as well as in the hippocampal formation. This ‘remaining’ spatial map is most strongly associated with the following terms in the literature: ‘default mode’, ‘autobiographical’, ‘episodic’, ‘self-referential’ and ‘social’. c) Subtraction of the mtDMN^+^ from the dmPFC DMN^+^ reveals remaining above-average activation in the temporoparietal junction, the lateral temporal cortex, anterior and dorsomedial prefrontal cortex and posterior cingulate cortex. This ‘remaining’ spatial map is most strongly associated with the following terms in the literature: ‘default mode’ ‘self-referential’, ‘autobiographical’, ‘theory of mind’, and ‘mentalising’. The mentioned associations are in line with cognitive functions already ascribed to the mtDMN^+^ (contextualised retrieval of episodic memories)^47^ and the dmPFC DMN^+^ (mentalizing and theory of mind)^74^; and with another study using the same automatic meta-analysis tool developed by*^63,71^ *to relate DMN spatial maps to cognitive functions^50^.*


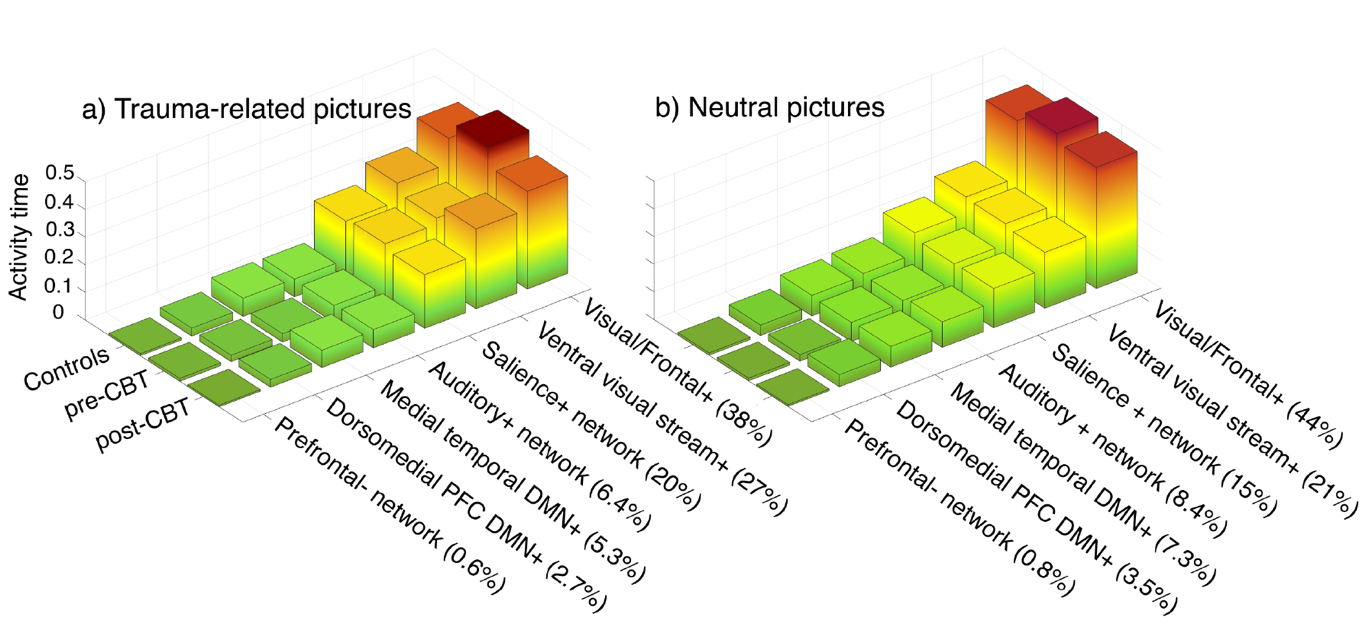


Supplementary figure 4. Healthy controls and patients with PTSD before and after CT spend different amounts of time on each network in the trauma-related and neutral conditions. Activation time for each of the networks is more consistent across groups in neutral (b) than in the trauma-related (a) condition. Furthermore, the smaller percentage of time occupied by activation of the Visual/Frontal^+^ network during presentation of trauma-related pictures (a) in contrast to neutral pictures (b) might be compensated through higher activation times of the salience^+^ network and the ventral visual stream^+^.

***
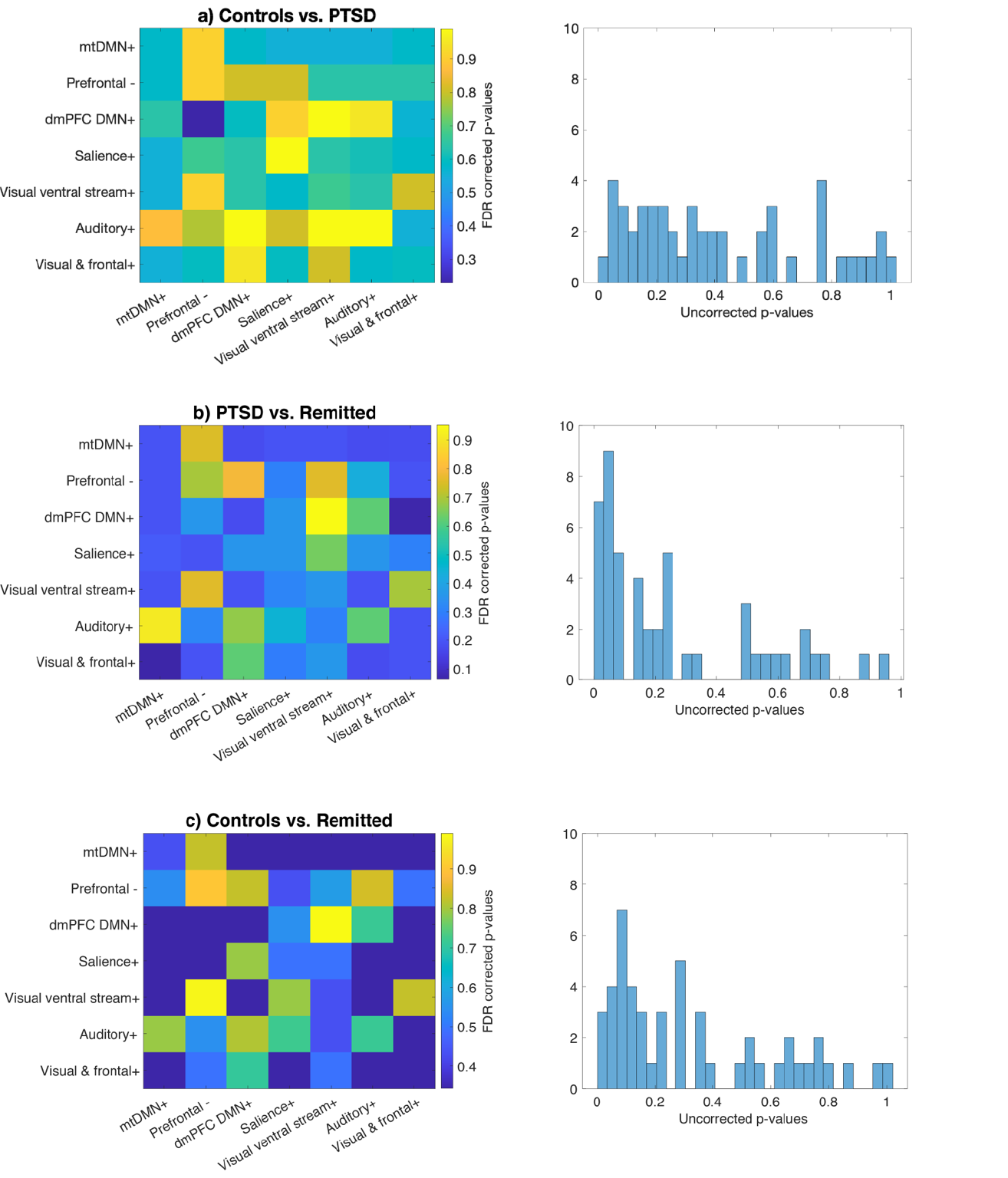
***

***Supplementary figure 5. No significant differences were observed between controls, PTSD and remitted participants in states transitions.*** *Matrix of p-values for the states’ transition corrected for multiple comparisons (**FWER < 0.05) (left) and histogram of uncorrected p-values (right). After correcting for multiple comparisons, no significant differences were observed any of the states (left, a, b and c). Results before correcting for multiple comparisons (right) indicate that there might be trend towards significance when comparing PTSD vs. remitted and controls vs. remitted (b and c), since the histogram of p-values is not uniformly-distributed but peaks on the left side.*

***
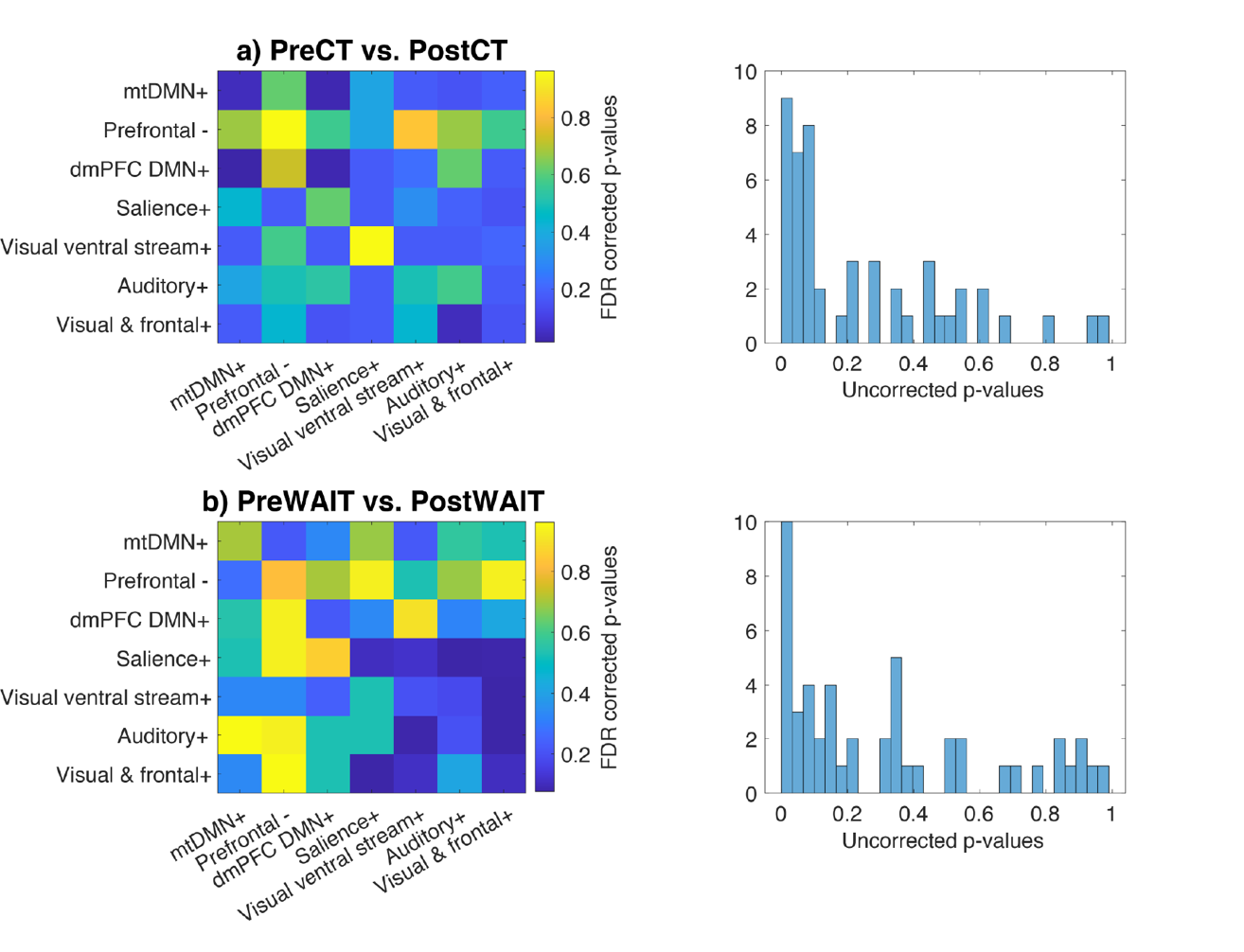
***

***Supplementary figure 6****.* ***Differences in the number of transitions between DMN subnetworks are observable before and after CT, but not before and after being on a waiting list****. Matrix of p-values for the states’ transition corrected for multiple comparisons (**FWER < 0.05) (left) and histogram of uncorrected p-values (right). a) Significant differences were observed in participants before and after CT in the transitions between the two DMN subnetworks (mtDMN^+^ and dmPFC DMN^+^). b) No significant differences were observed between any of the states when comparing participants before and after being on a waiting list; but again there is a trend towards significance, given the non-uniform distribution of the histogram of p-values****.***
